# Analysis of Corneal Distortion after Myopic PRK

**DOI:** 10.3390/jcm10010082

**Published:** 2020-12-28

**Authors:** Michele Lanza, Luigi De Rosa, Sandro Sbordone, Rosa Boccia, Ugo Antonello Gironi Carnevale, Francesca Simonelli

**Affiliations:** Multidisciplinary Department of Medical, Surgical and Dental Sciences, University of Campania Luigi Vanvitelli, 80132 Napoli, Italy; luigiderosa@gmail.com (L.D.R.); sandro.omar65@gmail.com (S.S.); rosa_boccia111@hotmail.com (R.B.); uagcce@alice.it (U.A.G.C.); francesca.simonelli@unicampania.it (F.S.)

**Keywords:** myopic PRK, corneal deformation, corneal biomechanical properties, ORA, Corvis ST

## Abstract

The purpose of the study is to evaluate the corneal biomechanical properties (CBP) and their behaviors after myopic refractive surgery both with Ocular Response Analyzer (ORA) and Corvis ST (CST). This retrospective study included 145 eyes of 145 patients with a mean age of 33.13 ± 9.24 years, who underwent myopic photorefractive keratectomy (PRK) for a refractive defect, measured as spherical equivalent, of mean −4.69 ± 2.04 D and have been evaluated before surgery and at 1, 3 and 6 months follow-up. Corneal hysteresis (CH) and corneal resistance factor (CRF) values significantly decreased after 1 month and remained statistically stable during further follow-ups. CST parameters had a different evolution: only second applanation time (AT2) differences showed a significant variation after 1 month that did not statistically change over time. Highest concavity deformation amplitude (HCDA), highest concavity peak distance (HCPD), first applanation time (AT1) and velocity (AV1) showed continuous significant differences both after 3 and after 6 months. This study suggests that after central surface ablation surgery, such as myopic PRK, corneal shape is remodeling, and its deformation parameters are going to change even at 6 months follow-up. This indicates that it should be important to evaluate refractive surgery patients during a longer follow-up because this could allow earlier diagnosis and better management of late-onset complications.

## 1. Introduction

Assessment of in vivo corneal biomechanical properties (CBP) is gaining more importance in many aspects of ophthalmology, such as refractive surgery [1], corneal transplant [2,3], intraocular pressure evaluation [4] and overall eyeball deformation analysis [5]. There are currently two devices able to provide CBP evaluation, and both of them analyze the corneal response to an air pulse stimulus: the Ocular Response Analyzer (ORA; Reichert Ophthalmic Instruments, Buffalo, NY, USA) [6] and the Corvis ST (CST; Oculus, Wetzlar, Germany) [7,8]. A better knowledge of the overall process of both corneal and eyeball deformations could lead to better understanding the reasons of corneal response both to external stimuli, such as surgically induced ones, and internal ones, such as those determined by inner degeneration such as keratoconus [4]. CBP are largely investigated in many types of eyes, aiming mainly to provide better indications for diagnosis and management of corneal diseases or better screening for corneal surgery [4,9]. One of the most important fields of application of CBP evaluation is the refractive surgery [10]. Photorefractive keratectomy (PRK) is the first technique, introduced in the late 1990s, designed to change corneal shape to treat refractive defects [11]. Despite the further development of other techniques, able to obtain very good results, such as laser in situ keratomileusis (LASIK), laser-assisted subepithelial keratomileusis (LASEK), laser in situ keratomileusis (EPI-LASIK), small incision lenticule extraction (SMILE) and femtosecond laser-assisted LASIK (FS-LASIK), PRK remains one of the most performed and successful kinds of refractive surgery thanks to innovations in diagnostic devices, in ablation strategy designs and in excimer laser developments [11]. Even if some of the other mentioned procedures are also accepted, PRK is the preferred choice to correct myopia in the Air Forces of various countries, including the US, in which a very high level of quality of vision and stable results are required [12,13]. Moreover, PRK has been proven to be safe and effective also in correction of high myopic defects [14]. Although other studies about CBP evaluation after refractive surgery have been previously published, unanimous conclusions have not been drawn. Moreover, CBP changes induced by PRK technique have not been extensively studied, the limits of most of the previously published papers are related to the size of the sample, the duration of the follow-up, the lack of comparison between ORA and CST and/or an actual deep analysis of the results observed [1,10,15,16]. The purpose of this study is to provide an accurate and detailed analysis of CBP changes measured with both ORA and CST devices in a fair sample of eyes before and after undergoing myopic PRK.

## 2. Materials and Methods

This retrospective study included 145 eyes of 145 patients that underwent myopic PRK with a mean age of 33.13 ± 9.24 years (range from 19 to 55 years). Before surgery, the mean refractive error, measured as spherical equivalent, ranged from −10.25 to −0.50 D (mean −4.69 ± 2.00 D).

Exclusion criteria for this study were all systemic and/or ocular conditions that could interfere with the corneal healing process, refractive outcome, or intra-ocular pressure (IOP) measurement, such as diabetes, dry eye, uveitis, connective tissue disorders, corneal or lens opacities.

The study followed the tenets of the 1964 Declaration of Helsinki and was approved by the local clinical research ethics committee (approval code 0014459/i), and informed consent was obtained from all patients before examination and before surgery.

Myopic PRK was performed under topical anesthesia using oxybuprocaine eye drops (Benoxinato, Alfa Intes, Casoria, Italy). The lids were opened with a speculum, the epithelium was debrided with a mechanical brush, and the treatment was performed with a Wave-Light Allegretto Wave Eye-Q 400 Hz excimer laser (WaveLight GmbH, Alcon, Fort Worth, TX, USA). Mitomicin-C was never used in the cases included in this study. A bandage contact lens was applied under sterile conditions on the treated eye immediately after surgery and was left until complete re-epithelialization. During this time lapse, the operated eye received the following medications: diclofenac sodium 0.1% eye drops twice a day for the first 2 days, nethylmicin preservative-free eye drops until re-epithelialization and preservative-free artificial tears for 1 month; after re-epithelialization, clobetasone eye drops were prescribed to all patients for 1 month, 4 times a day.

Pre-operative and follow-up examinations at 1, 3 and 6 months after PRK, included a comprehensive ophthalmologic examination, intra-ocular pressure (IOP) evaluation with Goldmann applanation tonometry (GAT), Oculus Pentacam (Oculus, Wetzlar, Germany) scan and CBP analysis both with ORA and with CST. ORA and CST devices were used by two different physicians, each unaware of the other results, while a third physician collected and analyzed all the data.

None of the eyes included in this study developed corneal haze or other complications related to refractive surgery during the considered follow-up.

Oculus Pentacam is a corneal tomographer using a rotating Scheimpflug camera and a monochromatic slit light source (blue led at 475 nm) that rotate together around the optical axes of the eye to calculate a three-dimensional model of the anterior segment, including data from anterior and posterior corneal topography, pachymetry and measurements of anterior chamber depth, lens opacity and lens thickness. Within 2 s, the system rotates by 180° and acquires 25 or 50 images (depending on the settings of the user) that contain 500 measurement points on the front and back corneal surfaces to draw a true elevation map [17,18]. For this study, the 25 images per scan option was chosen. The parameters evaluated were: central corneal thickness (CCT) at pupil center and anterior corneal curvature measured with Sim’K (MK).

Corvis ST is a device that uses a 4330 images per second Scheimpflug camera to record the corneal behavior during an air puff indentation. It provides many different parameters related to two main moments: the first applanation (applanation time AT1, applanation length AL1, applanation velocity AV1) when the cornea flattens because of the air puff, and the second applanation (applanation time AT2, applanation length AL2, applanation velocity AV2), when the cornea flattens again after reaching the highest concavity (highest concavity time (HCT), highest concavity peak distance (HCPD), highest concavity peak radius (HCPR), highest concavity deformation amplitude (HCDA)) shape in order to return to its original state. In this study, each parameter provided by CST was included in the analysis [4,19].

ORA (Reichert, Inc., Depew, NY, USA) is a device able to provide a measurement of IOP through compensation for corneal properties. It is able to record IOP, detecting the amount of infrared reflex during the applanations occurring when the cornea is moving both inward and outward, returning to the original position. The difference between the values obtained in the two different moments is called corneal hysteresis (CH) and reflects the viscoelastic nature of the cornea. Corneal Resistance Factor (CRF), the second parameter provided by the ORA, is strongly related to corneal thickness and provides information about the corneal static resistance [20].

### Statistical Analysis

The normality of the distribution of the study population was analyzed with the Kolmogorov–Smirnov test. Analysis of differences and data correlations not reaching normality standards were performed using non-parametric tests. Particularly, a Friedman test, as a non-parametric alternative to analysis of variance (ANOVA), was performed, followed by a post-hoc Wilcoxon signed rank test to evaluate comparisons among values obtained at different times and by different instruments.

Furthermore, all correlations have been evaluated using non-parametric (Spearman) tests. Level of significance was set at *p* < 0.05 for all statistical tests after study population analysis and error evaluation of the tested devices. Statistical evaluations were performed using SPSS software (IBM Corp. Armonk, New York, NY, USA) version 18.0. Even though all patients underwent bilateral evaluation and bilateral surgery, only the right eye data was considered in the statistical analysis to eliminate any potential bias related to the inner correlations between pair organs of the same subject.

## 3. Results

The means and standard deviations of refractive errors, CCT, MK and IOP measured before surgery and at 1, 3 and 6 months follow-up are summarized in Table 1. The means of CBP evaluated both with ORA and CST, measured before surgery and at 1, 3 and 6 months follow-up, are summarized in Table 2. No correlations between age and changes in any of the CBP parameters evaluated were observed. As shown in Table 2, CH and CRF values significantly decreased after 1 month and they remained statistically stable over time. Changes detected in CST parameters had a different behavior: only AT2 showed a significant change after 1 month that did not statistically change during further follow-up, whereas AT1, AV1, HCPD and HCDA showed a continuous significant change both after 3 and after 6 months (Table 2). Moreover, most of the other CST features analyzed had shown significant changes at 3 months follow-up compared to 1 month values.

Analyzing the correlations between CBP parameters and other features at 6 months follow-up, it is possible to observe that both CH and CRF showed significant correlations with effective treatment, CCT and MK differences. Moreover, a significant direct correlation was observed between CRF changes and GAT variations. Interestingly, changes in morphological parameters such as CCT and MK had not shown significant correlations with CBP parameters provided by CST, whereas GAT differences appeared to be correlated with many of them (Table 3).

## 4. Discussion

Refractive surgery is one of the most important fields of ophthalmology, given the ever-increasing demand of patients requesting it. This leads physicians to always look for better results, developing new techniques and pushing for improving the quality of both diagnostic and treatment devices [21]. Whereas new techniques such as FemtoLASIK or SMILE have been recently introduced, their superiority to surface ablation strategies, such as PRK or LASEK, has not yet been proven [21,22,23].

Thus, it is important to evaluate the effect that this kind of surgery could determine on the overall corneal deformation properties. It could help to identify the ranges of parameters of eyes that could undergo refractive treatment without developing complications such as corneal ectasia, or to design new treatment strategies, more effective and less invasive for the cornea.

This is one of the first studies reporting differences in corneal deformation evaluation before and after myopic PRK with both devices currently available.

The results observed suggest that CST is able to detect more details in corneal deformation compared to ORA. In fact, while both CH and CRF decreased after 1 month from surgery and remained stable during further follow-up, parameters provided by CST showed more various behaviors. Some of them, such as AT1, AV1, HCPD and HCDA, had continuous changes during the overall follow-up time (Table 2).

One possible explanation of the different evolutions observed in these parameters after myopic PRK could be that they are provided by two different devices that work in different ways. Even if both of them evaluate the response of the cornea to an air puff stimulus, ORA detects it by two electro-optical systems able to measure the inward and outward applanation pressures, and thanks to these values, a dedicated algorithm processes them to obtain two final parameters: CH and CRF [20]. CST, on the other hand, evaluates the corneal distortion recording the deformation following an air puff stimulus with a high-definition Scheimpflug Camera [4,19]. Its software is able to provide many more parameters, related to speed, length and velocity of corneal deformations, giving more detailed and complex information related to the response of this tissue to external stimuli [4,19].

In this study, stronger correlations have been observed between changes in ORA parameters with CCT and MK differences compared to those detected between variations in CST features and the same corneal morphological variations (Table 3). This is particularly important because one of the main applications of corneal biomechanics in refractive surgery is to detect eyes that could be more likely to develop ectasia, improving the physicians’ ability to recognize them. Taking into account that physicians rely on this kind of screening mainly on morphological evaluations, such as corneal topography and tomography, to have biomechanical features highly connected to morphological ones does not appear as an actual improvement. Thus, these data support the increase of CST application together with corneal tomography in this field but also in keratoconus diagnostics [24].

The results observed in this study suggest that after central surface ablation surgery, such as myopic PRK, corneal shape is still remodeling, and its deformation parameters are going to change during the first 6 months after surgery.

Interestingly, even if most of the CST parameters showed a continuous change 3 months after surgery, those that did not stop their evolution at 6 months follow-up were related to the first applanation and to the highest concavity, but not the features related to the second applanation (Table 2). This suggests that myopic PRK could increase corneal deformability to external stimuli, producing a faster applanation (detected by AT1 and AV1) and an overall larger deformation (detected by HCPD and HCDA). The corneal ability to return to the original shape, on the other hand, seems to be stable 6 months after surgery.

This data could explain why sometimes a late-onset of corneal ectasia could occur or why a late regression of refraction correction could be observed, independently by the amount of the effective treatments. Moreover, these findings suggest that physicians should plan a longer follow-up to check their refractive surgery patients, to avoid delayed complication detections. An early diagnosis could, indeed, allow a better management and a better final resolution of eventual long-term complications.

The results obtained here agree with those published by Yu et al. [25] and by Chen et al. [17], but here, the analyses of CBP performed with CST in eyes undergoing refractive surgery have been added. Thus, this study is able to provide more information about the overall corneal deformation after surface ablation surgery during more detailed follow-up.

Hashemi et al. [26] have published an interesting paper evaluating corneal deformation in patients undergoing myopic FemtoLASIK and myopic PRK, but they measured CBP only with CST. The findings by Hashemi et al. mostly agree with the current ones but they observed a reduction of AV1, whereas in our study, an increase has been detected [26]. This could be explained by several factors, such as the different study population (145 eyes vs. 30) or the differences in the myopic defect treated (in this study, even defects higher than 7D underwent surgery) [26]. Moreover, it is difficult to imagine that time and velocity of deformation are both reduced because these two parameters are inversely connected.

The data included in this study could also be useful as a range of CBP parameters of eyes that did not develop corneal ectasia after myopic PRK, and this could help in detection of suspect cases both pre-operatively and post-operatively.

Among the limits of the present study, it is possible to include the sample size and the follow-up that should be increased in further research; moreover, here, no complications such as corneal ectasia have been observed, so further investigations are needed to have information about these kind of eyes.

## 5. Conclusions

In conclusion, even if further studies overcoming the listed limitations are needed to completely understand the complex mechanisms of corneal shape modification after surface ablation, the results observed suggest that CST could perform a corneal deformation analysis less affected by morphological parameters like CCT, MK or effective treatment in eyes that have undergone myopic PRK. Moreover, it appears that corneal shape modifications are still active during the first months after surgery and CST is able to detect them, showing the changes in some of the corneal deformation parameters until 6 months follow-up. A deeper study of these features could help to make an early diagnosis of late onset post-operative complications and to improve the accuracy of the screening of these patients.

## Figures and Tables

**Table 1:** The means ± standard deviations of refractive errors (SE), central corneal thickness (CCT), mean keratometry (MK) and intra-ocular pressure (IOP) measured before photorefractive keratectomy (PRK) and at 1, 3 and 6 months of follow-up (FU).

	Baseline	1 Month FU	*p*	3 Months FU	*p*	6 Months FU	*p*
SE (D)	−4.69 ± 2.04	0.06 ± 0.57	*<0.01*	0.01 ± 0.73	**<0.01**	0.04 ± 0.6	*<0.01*
CCT (µm)	560.49 ± 30.94	487.9 ± 42.79	*<0.01*	485.15 ± 43.43	**<0.01**	484.17 ± 42.87	*<0.01*
MK (D)	43.58 ± 1.46	39.68 ± 2.07	*<0.01*	39.82 ± 2.05	**<0.01**	39.73 ± 1.95	*<0.01*
IOP (mmHg)	14.81 ± 2.37	14.36 ± 2.26	*<0.01*	13.24 ± 2.16	**<0.01**	12.73 ± 1.8	*<0.01*

**Table 2:** The means ± standard deviations of corneal biomechanical properties (CBP) evaluated both with ORA Ocular Response Analyzer (ORA) and Corvis ST (CST), measured before photorefractive keratectomy (PRK) and at 1, 3 and 6 months follow-up (FU).

	Baseline	1 Month FU	Baseline vs. 1 Month FU (*p*)	3 Months FU	Baseline vs. 3 Months FU (*p*)	1 Month vs. 3 Months FU (*p*)	6 Months FU	Baseline vs. 6 Months FU (*p*)	3 Months FU vs. 6 Months FU (*p*)
CH (mmHg)	10.21 ± 1.72	8.08 ± 1.85	*<0.01*	8.16 ± 1.84	*<0.01*	0.161	8.31 ± 1.69	*<0.01*	0.199
CRF (mmHg)	10.51 ± 2.03	7.8 ± 2.2	*<0.01*	7.63 ± 2.02	*<0.01*	0.246	7.61 ± 1.86	*<0.01*	0.905
AT1 (ms)	7.38 ± 0.52	7.34 ± 0.6	*<0.01*	7.12 ± 0.55	*<0.01*	*<0.01*	7.08 ± 0.54	*<0.01*	*<0.01*
AL1 (mm)	1.74 ± 0.26	1.72 ± 0.26	*0.025*	1.76 ± 0.27	0.337	*<0.01*	1.76 ± 0.29	0.341	0.532
AV1 (m/s)	0.15 ± 0.03	0.15 ± 0.03	*0.011*	0.16 ± 0.03	*<0.01*	*<0.01*	0.17 ± 0.03	*<0.01*	*0.024*
AT2 (ms)	21.37 ± 0.43	21.71 ± 5.05	*<0.01*	21.76 ± 5.04	0.331	0.293	21.84 ± 5.03	0.189	0.275
AL2 (mm)	1.87 ± 0.45	1.84 ± 0.46	*<0.01*	1.97 ± 0.49	*0.014*	*<0.01*	1.99 ± 0.49	*0.014*	0.169
AV2 (m/s)	−0.34 ± 0.09	−0.37 ± 0.11	0.383	−0.37 0.1	*<0.01*	*<0.01*	−0.38 ± 0.10	*<0.01*	0.109
HCT (ms)	16.51 ± 0.57	16.4 ± 0.64	*<0.01*	16.29 ± 0.65	*<0.01*	*<0.01*	16.31 ± 0.64	*<0.01*	0.575
HCPD (mm)	4.68 ± 0.31	4.7 ± 0.35	0.714	4.77 ± 0.32	*<0.01*	*0.010*	4.84 ± 0.27	*<0.01*	*<0.01*
HCPR (mm)	7.02 ± 0.91	6.98 ± 0.93	0.651	6.78 ± 0.9	*0.011*	*0.047*	6.71 ± 0.87	*<0.01*	0.618
HCDA (mm)	1.02 ± 0.12	1.2 ± 0.88	*<0.01*	1.09 ± 0.11	*<0.01*	*<0.01*	1.1 ± 0.12	*<0.01*	*0.024*

CH: Corneal hysteresis, CRF: Corneal resistance factor, AT1: First applanation time, AL1: First applanation length, AV1: First applanation velocity, AT2: Second applanation time, AL2: Second applanation length, AV2: Second applanation velocity, HCT: Highest concavity time, HCPD: Highest concavity peak distance, HCPR: Highest concavity peak radius, HCDA: Highest concavity deformation amplitude.

**Table 3:** Correlations between corneal biomechanical properties (CBP) parameters and other features at 6 months follow-up.

		CH	CRF	AT1	AL1	AV1	AT2	AL2	AV2	HCT	HCPD	HCPR	HCDA
Effective treatment	Correlation Coefficient	−0.290	−0.331	0.057	0.103	−0.020	−0.224	−0.083	0.107	−0.040	−0.093	0.069	−0.175
	*p*	*<0.01*	*<0.01*	0.497	0.216	0.809	*0.007*	0.320	0.198	0.631	0.266	0.411	*0.036*
CCT	Correlation Coefficient	0.401	0.460	0.008	−0.082	−0.071	0.140	0.076	−0.147	−0.015	0.094	−0.055	0.083
	*p*	*<0.01*	*<0.01*	0.925	0.326	0.397	0.094	0.361	0.078	0.857	0.263	0.513	0.322
GAT	Correlation Coefficient	0.095	0.187	0.438	−0.036	−0.111	−0.339	0.189	0.188	−0.105	−0.235	0.221	−0.352
	*p*	0.258	*0.024*	*<0.01*	0.669	0.184	*<0.01*	*0.023*	*0.024*	0.210	*<0.01*	*<0.01*	*<0.01*
MK	Correlation Coefficient	0.252	0.292	−0.022	−0.061	0.075	0.152	−0.019	−0.059	0.112	0.026	−0.094	0.128
	*p*	*0.002*	*<0.01*	0.794	0.466	0.370	*0.068*	0.819	0.484	0.181	0.754	0.259	0.125

CCT: Central Corneal Thickness, MK: Mean Keratometry, GAT: Goldmann applanation tonometry, CH: Corneal Hysteresis, CRF: Corneal Resistance Factor, AT1: First Applanation Time, AL1: First Applanation Length, AV1: First Applanation Velocity, AT2: Second Applanation Time, AL2: Second Applanation Length, AV2: Second Applanation Velocity, HCT: Highest Concavity Time, HCPD: Highest Concavity Peak Distance, HCPR: Highest Concavity Peak Radius, HCDA: Highest Concavity Deformation Amplitude.

## Data Availability

The data presented in this study are available on request from the corresponding author. The data are not publicly available due to privacy and ethical reasons.
